# Mechanisms of Action and Efficacy of Hyaluronic Acid, Corticosteroids and Platelet-Rich Plasma in the Treatment of Temporomandibular Joint Osteoarthritis—A Systematic Review

**DOI:** 10.3390/ijms22147405

**Published:** 2021-07-09

**Authors:** Marcin Derwich, Maria Mitus-Kenig, Elzbieta Pawlowska

**Affiliations:** 1 ORTODENT, Specialist Orthodontic Private Practice in Grudziadz, 86-300 Grudziadz, Poland; 2 Department of Experimental Dentistry and Prophylaxis, Medical College, Jagiellonian University in Krakow, 31-008 Krakow, Poland; maria.mitus@interia.pl; 3 Department of Orthodontics, Medical University of Lodz, 90-419 Lodz, Poland; elzbieta.pawlowska@umed.lodz.pl

**Keywords:** temporomandibular joint osteoarthritis, hyaluronic acid, corticosteroids, platelet-rich plasma, temporomandibular joint disorders, arthrocentesis, intraarticular injections

## Abstract

Temporomandibular joint osteoarthritis (TMJ OA) is a low-inflammatory disorder with multifactorial etiology. The aim of this review was to present the current state of knowledge regarding the mechanisms of action and the efficacy of hyaluronic acid (HA), corticosteroids (CS) and platelet-rich plasma (PRP) in the treatment of TMJ OA.: The PubMed database was analyzed with the keywords: “(temporomandibular joint) AND ((osteoarthritis) OR (dysfunction) OR (disorders) OR (pain)) AND ((treatment) OR (arthrocentesis) OR (arthroscopy) OR (injection)) AND ((hyaluronic acid) OR (corticosteroid) OR (platelet rich plasma))”. After screening of 363 results, 16 studies were included in this review. Arthrocentesis alone effectively reduces pain and improves jaw function in patients diagnosed with TMJ OA. Additional injections of HA, either low-molecular-weight (LMW) HA or high-molecular-weight (HMW) HA, or CS at the end of the arthrocentesis do not improve the final clinical outcomes. CS present several negative effects on the articular cartilage. Results related to additional PRP injections are not consistent and are rather questionable. Further studies should be multicenter, based on a larger group of patients and should answer the question of whether other methods of TMJ OA treatment are more beneficial for the patients than simple arthrocentesis.

## 1. Introduction

According to the Diagnostic Criteria for Temporomandibular Disorders (DC/TMD), there have been listed twelve different types of temporomandibular disorders (TMD), including: myalgia, local myalgia, myofascial pain, myofascial pain with referral, arthralgia, headache attributed to TMD, disc displacement with reduction, disc displacement with reduction with intermittent locking, disc displacement without reduction with limited opening, disc displacement without reduction without limited opening, degenerative joint disease and subluxation [1].

TMJ arthritic conditions have been subdivided into two groups, namely low-inflammatory and high-inflammatory disorders [2]. Osteoarthritis (OA) and post-traumatic arthritis have been classified as low-inflammatory disorders, whereas rheumatoid arthritis, metabolic arthritic diseases (i.e., gout, pseudogout, lupus erythematosus) and infectious arthritis have been classified as high-inflammatory disorders [2]. The general characteristics for low-inflammatory disorders encompass: the involvement of one or both TMJs, the presence of localized pain and the presence of TMJ crepitation. TMJ clicking and the presence of the rheumatological factor are rare, the erythrocyte sedimentation rate (ESR) is often normal, and the cyclic citrullinated peptide antibody (CPP) is normal; however, the concentration of the C-reactive protein (CRP) may be elevated [2]. Contrary to the low-inflammatory disorders, the high-inflammatory disorders are characterized by bilateral involvement of TMJs, diffused pain, lack of clicking, rare occurrence of crepitation, presence of rheumatoid factor, elevated ESR, CPP and CRP [2].

Temporomandibular joint osteoarthritis (TMJ OA) is considered to be a combination of degenerative joint disease and joint pain [1]. It is a disease involving an entire joint [3,4]. The etiology of TMJ OA is multifactorial [5,6]. We have described it thoroughly in our previous manuscripts [7,8]. Because of the fact that the TMJ OA etiological factors are very complex, the treatment of TMJ OA requires a multidisciplinary approach [7,8,9]. There have been listed three major aims for the treatment of the TMJ OA, namely TMJ pain reduction, reestablishment of the normal mandibular movements, as well as the improvement of the patients’ quality of life [10]. Although there are several different methods of treatment of TMJ OA, none of them is unequivocally the most effective one [10]. The most popular are noninvasive, conservative methods of treatment, including physiotherapy, occlusal splint therapy and pharmacotherapy. Less invasive surgical procedures used in the treatment of the TMJ OA encompass intraarticular injections of HA, CS or growth factors, arthrocentesis alone and finally the combination of arthrocentesis and intraarticular injections. Finally, the invasive surgical procedures comprise arthroscopy and open joint surgeries, including, among others, discectomy, high condylectomy and arthroplasty [7,8,9,10].

The aim of this review was to present the current state of knowledge regarding the efficacy of HA, CS and PRP in the treatment of TMJ OA on the basis of the literature.

## 2. Materials and Methods

### 2.1. Clinical Question

What is the efficacy of hyaluronic acid (HA), corticosteroids (CS) and platelet-rich plasma (PRP) in the treatment of TMJ OA in humans on the basis of the literature?

### 2.2. Inclusion and Exclusion Criteria

Table 1 presents inclusion and exclusion criteria for the systematic review.

### 2.3. The PICO Approach

We used the PICO approach to properly develop literature search strategies for this review:

Population:

Adolescents (aged: 16 years old or more) and adult patients who were diagnosed with TMJ OA.

Intervention:

Temporomandibular joint arthrocentesis or arthroscopy with an additional injection of HA, CS and PRP or intraarticular injections of HA, CS and PRP in patients diagnosed with TMJ OA.

Comparison:

Intraarticular injection of HA, CS or PRP, arthrocentesis or arthroscopy with an additional injection of HA, CS or PRP, arthrocentesis or arthroscopy alone, placebo; randomized controlled trials (RCTs) and randomized clinical trials were included in the review. 

Outcome:

Decreased pain in the temporomandibular joint area and increased maximum mouth opening.

### 2.4. Search Strategy

The PubMed database was analyzed with the following keywords: (temporomandibular joint) AND ((osteoarthritis) OR (dysfunction) OR (disorders) OR (pain)) AND ((treatment) OR (arthrocentesis) OR (arthroscopy) OR (injection)) AND ((hyaluronic acid) OR (corticosteroid) OR (platelet rich plasma)). After screening of 363 results, 16 studies were included in this review. We included RCTs and randomized clinical trials in this review.

**Figure 1:**
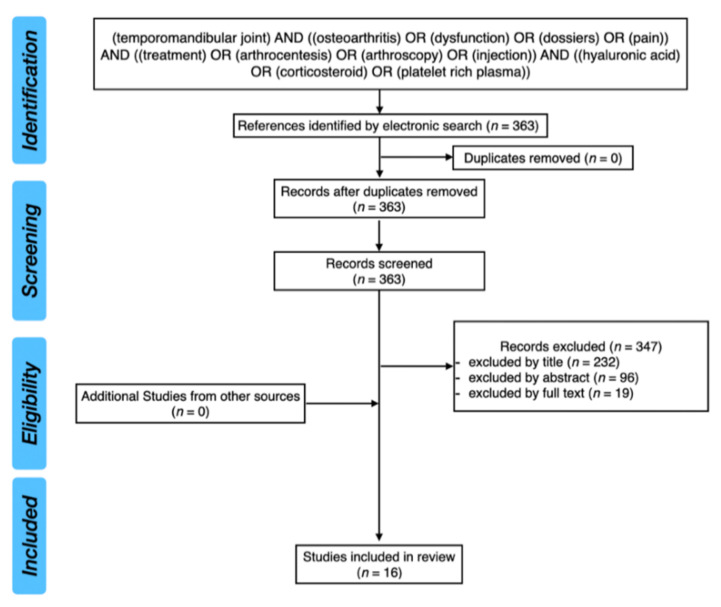
PRISMA flow diagram for review of the literature.

Figure 1 presents the PRISMA flow diagram for a review of the literature.

### 2.5. Cohen’s Kappa Coefficient

Cohen’s kappa coefficient between the reviewers was of 1.00.

## 3. Results and Discussion

### 3.1. Hyaluronic Acid (HA)

HA is a nonsulfated glycosaminoglycan, a polysaccharide, made up of repeated units of D-glucuronic acid and N-acetylglucosamine with alternating beta (1–3) glucuronide and beta (1–4) glucosaminidic bonds. HA physiologically occurs within the articular cartilage and the synovial fluid [11,12,13,14,15,16]. It is synthesized by fibroblast-like cells, known as the synoviocytes type B [14,17]. There exist three isoforms of hyaluronan synthases in humans, namely HAS1, HAS2 and HAS3 [18,19]. They are integral membrane proteins [19]. HAS1 and HAS2 are responsible for the polymerization of HA chains up to 2000 kDa, whereas HAS3 polymerizes shorter HA chains of the length of 200–300 kDa [20].

**Figure 2:**
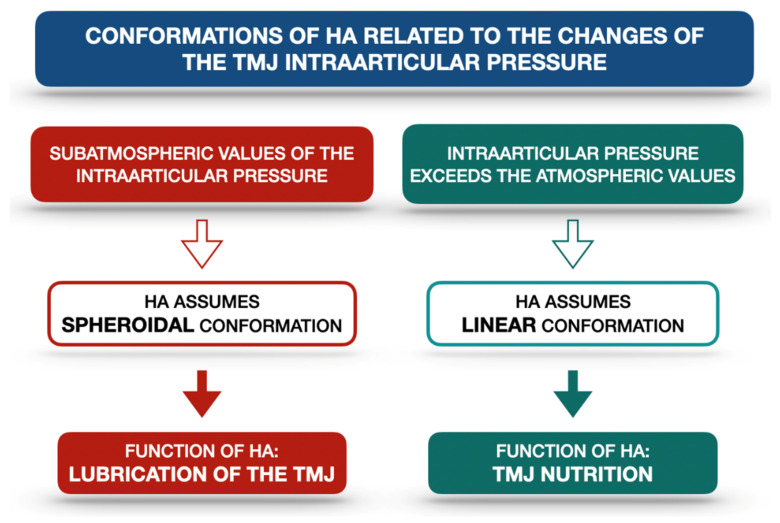
Schematic relationship between the TMJ intraarticular pressure and HA conformations on the basis of the literature [14]. HA—hyaluronic acid, TMJ—temporomandibular joint.

HA forms a layer which not only covers but also penetrates the articular surfaces. It is combined with different proteins, coming from the synovial fluid [14]. There are two conformations in which HA occurs: the linear and the spheroidal ones [14]. It has been proven that HA plays a significant role in the nutrition and lubrication of the TMJ articular surfaces [13,14,16]. This role is directly related to the value of the intraarticular pressure, which makes the HA change its conformation. When the intraarticular pressure reaches subatmospheric values, the proteins lose contact with the articular surfaces and the HA assumes a spheroidal conformation, allowing sliding movements within the TMJ. When the intraarticular pressure exceeds atmospheric values, the HA occurs in the linear form and penetrates the fibrocartilage, which is necessary for TMJ nutrition [14]. Moreover, HA is found to stabilize all of the TMJ components [14]. Figure 2 presents the schematic relationship between the TMJ intraarticular pressure and HA conformations on the basis of the literature [14].

Although the HA lubricates the TMJ articular surfaces, it must be emphasized that HA by itself does not reduce the intraarticular friction satisfactorily (coefficient of friction ca. μ ≈ 0.3) [11,21,22,23]. There are three molecules, HA, lubricin (proteoglycan) and phosphatidylcholine lipids (phospholipids), that form a boundary lubrication layer, which leads to a significant reduction in friction within the joint (coefficient of friction down to μ ≈ 0.001 at pressures over 100 atm) [21]. It has been found that lubricin, localized in the superficial zone, anchors the HA chains at the outer surface of the articular cartilage. Therefore, HA becomes attached to the surface and consequently complexes with phosphatidylcholines. Highly hydrated phosphocholine groups become exposed between the opposite cartilage surfaces. This connection forms a boundary lubrication layer [21]. It is also possible for the HA from the synovial fluid to attach simultaneously to the lipids localized on the opposite cartilage surfaces. Surprisingly, these polymer bridges do not increase the friction between the articular surfaces [11]. Lin et al. [11] presented two possible mechanisms which could have explained the observed data. First of all, there are lipid multilayers on each articular surface. The HA polymer bridges are localized on the midplane. Therefore, to eliminate the increased friction, the slip plane is moved from the midplane (which is full of HA bridges) to the interface within one of the lipid multilayers (free of HA). Secondly, lipids from the synovial fluid do interact with the free HA. As a consequence, the number of HA polymer bridges between the articular surfaces becomes reduced [11].

The HA molecular weight influences the viscoelastic properties of the synovial fluid. High-molecular-weight HA (HMW HA), which is more than 1000 kDa, contributes to the viscoelasticity of the synovial fluid [20]. Iturriaga et al. [24] distinguished three different categories of the exogenous HA preparations regarding their molecular weight, namely low-molecular-weight HA (LMW HA) 500–1000 kDa, medium-molecular-weight (MMW HA) 1200–4500 kDa and HMW HA 6000–7000 kDa. HMW HA presents an anti-inflammatory effect [25]. According to the study by Herzog et al. [26], HMW HA controls the hydrodynamics (viscosity, compressive stiffness and elasticity) of the synovial fluid via an entropy-driven excluded volume effect.

Hyaluronidases are the enzymes which are responsible for the degradation of HMW HA into LMW HA [20]. LMW HA presents proinflammatory properties, which are manifested by the induction of macrophage genes’ expression [27]. There have been found six different hyaluronidase-like genes within the human genome [28]. However, there are only two major hyaluronidases (HYAL1 and HYAL2) in human somatic tissues [18,28]. The process of HA degradation is associated with aging, inflammation and is also observed in the course of osteoarthritis. It has also been described that the reactive oxidative radical species may inhibit the HA biosynthesis, as well as lead to the depolymerization of the already biosynthesized HA chains [6,29]. The reactive oxidative radical species are released within the TMJ due to the repetitive cycles of temporary hypoxia and re-oxygenation [6]. TMJ mechanical overloading affects the HA metabolism, leading to condylar cartilage degradation [18]. The increased amount of LMW HA is the direct cause of decreased synovial fluid viscosity. As a consequence, the friction between the articular surfaces increases and the articular surfaces become progressively damaged [20]. Takahashi et al. [30] found that a group of patients diagnosed with TMD (internal derangements and OA) presented HA of a significantly lower molecular weight in synovial fluid compared to healthy controls. Guo et al. [31] performed a study in a group of growing rats and confirmed that TMJ mechanical overloading affects the HA metabolism. They found that the functional lateral shift of the mandible stimulated the expression of HYAL1 and HYAL2 in both TMJs. Therefore, the functional lateral shift of the mandible changed the lubrication of the TMJs [31]. 

According to the recent research, there has been confirmed a relationship between chronic hypoxia, increased HYAL-1 plasma concentration and increased HMW HA degradation. These changes may enhance systemic inflammation in the course of obstructive sleep apnea [32].

Because of the increased amount of LMW HA within osteoarthritic TMJs, some have suggested the use of HMW HA in the treatment of TMJ OA. Tolba et al. [33] observed that intraarticular injections of HMW HA led to a satisfactory reduction in osteoarthritic changes within the TMJs. Similar observations were presented by Duygu et al. [34]. Lemos et al. [35] concluded that HMW HA may have a positive impact on osteoarthritic TMJs, because in individuals treated with HMW HA, the authors observed, among others, limited histologic changes, lower activity of metalloproteinases (MMP-2 and MMP-9), as well as a greater arrangement of collagenous fibers. Contrary to the previously mentioned studies, Iturriaga et al. [24] compared the efficacy of LMW HA and HMW HA intraarticular injections in the treatment of TMJ OA. The authors noticed that better results regarding the repairing processes of the cartilage and the articular disc were obtained with the usage of the LMW HA. Although the results presented by the above-listed authors [24,33,34,35] seem very promising, it must be emphasized that all of these studies were performed on different animal models, namely rats [33,35] and rabbits [24,34].

### 3.2. Corticosteroids (CS)

CS are hormones naturally occurring within the human body, which are biosynthesized by the adrenal cortex [36,37,38]. CS encompass both the glucocorticoids (i.e., cortisol) and mineralocorticoids (i.e., aldosterone) [36]. Glucocorticoids present principally anti-inflammatory and immunosuppressive effects, whereas mineralocorticoids regulate the ionic balance by stimulation of sodium reabsorption and potassium excretion [36,37].

There are several different methods to administer CS, including oral administration, aerosol for inhalation, topical administration, intravenous, intramuscular and finally intraarticular injections [36]. CS intraarticular injections have been used to treat different arthritic diseases (i.e., rheumatoid arthritis, osteoarthritis, gout) since 1951 [39].

**Figure 3:**
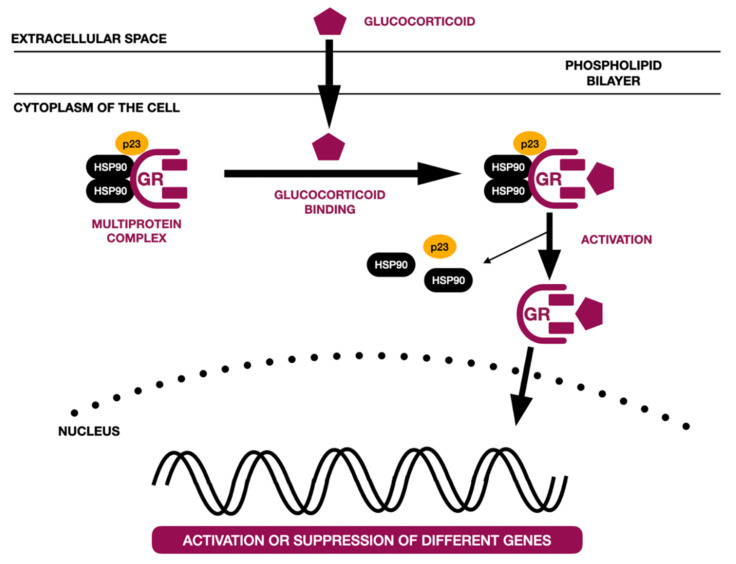
Schematic genomic action of glucocorticoid receptor on the basis of the literature [40,41]. GR—glucocorticoid receptor, hsp90 and p23—chaperone proteins.

There have been described different molecular models of glucocorticoid action [40,41,42,43,44,45]. The most common one, known as a genomic pathway, is associated with the activity of the glucocorticoid receptor (GR). The GR is localized within the cytoplasm and is part of the multiprotein complex. It is coupled with chaperone proteins and immunophilins. Glucocorticoids become bound to GRs and translocated to the nucleus. Within the nucleus, the GR directly affects (either activates or suppresses) the transcription of different genes by binding glucocorticoid response elements (GREs), by tethering itself to other transcription factors and affecting its activity, or in a composite manner (GR binds to half the GRE, which is near the binding site of another transcription factor) [40,41,42,43,44]. It is also possible for the GR to interact with different transcription factors, preventing their binding to DNA (transcription factor sequestration); to compete with other transcription factors for binding to DNA (competitive binding); and to compete with other transcription factors for cofactors necessary for transcription (co-factor competition) [43,44]. Figure 3 presents a schematic of the genomic action of the glucocorticoid receptor on the basis of the literature [40,41].

The second group of molecular mechanisms of glucocorticoid actions is known as the non-genomic pathway. Non-genomic action does not regulate gene expression; neither does it involve transcriptional processes or protein biosynthesis. Non-genomic action activates signal transduction pathways. It is based on the interactions between the glucocorticoids and the cell membrane (nonspecific interactions), as well as between the glucocorticoids and either cytosolic GRs or membrane-bound GRs [45]. There are four different mechanisms, described as non-genomic pathways, namely non-specific physicochemical interactions with membranes, chaperone protein signaling, mechanism via cell membrane receptors and finally a mechanism based on the competition for phosphoinositide 3-kinase. These mechanisms are not related to the direct combination of the glucocorticoid–GR complex with DNA [46].

Glucocorticoids stimulate the expression of annexin-1 (also known as lipcortin-1), which is the phospholipase A_2_ inhibitor. As a consequence, the biosynthesis of lipid mediators becomes inhibited, including the biosynthesis of prostaglandins, prostacyclin and leukotriene. Annexin-1 was also found to regulate, among others, cell proliferation and maturation, as well as neuroendocrine secretion [41,42]. Glucocorticoids also inhibit the transcription of many proinflammatory cytokines, including IL-1β, IL-6 and TNF-α. Therefore, glucocorticoids significantly suppress the inflammatory response [41,42].

There are several different formulations of CS to be injected intraarticularly, which have been accepted by the Food and Drug Administration (FDA) [38]. They can be allocated into one of the two subgroups, depending on the water solubility. Ester (acetate/acetonide) preparations are insoluble in water. They form microcrystalline particulates. Moreover, they present slower release and last longer at the site of injection [37,38]. Exemplary non-soluble CS are: methylprednisolone acetate, betamethasone acetate, hydrocortisone acetate and triamcinolone acetonide [37,38]. Contrary to the previously described group, non-ester preparation (sodium phosphate) formulations are nonparticulate and soluble in water. They begin working rapidly and, at the same time, the duration of their action is shorter. They do not form aggregates within the joint. Exemplary soluble CS are dexamethasone sodium phosphate and betamethasone sodium phosphate [37,38].

Table 2 presents examples of CS injected intraarticularly on the basis of the literature [37,38].

Intraarticular CS injections may cause several different types of local and systemic side effects. There have been listed the following local side effects: post-injection flare (the most common side effect), pain in the area of injection, subcutaneous trophy, skin depigmentation and soft tissue calcifications. Among the systemic adverse effects of intraarticular CS injection, there have been mentioned: facial flushing (related to the histamine-mediated response), hyperglycemic effects in patients suffering from diabetes (CS leads to insulin resistance), adrenal suppression and menstrual disturbances [36,37,38]. Moreover, the long-term use of CS leads to osteoporosis, due to the presence of bone catabolism and limited bone formation, associated with osteoblasts hypofunction and apoptosis [36,47].

Monseau et al. [48] presented a list of absolute and relative contraindications to musculoskeletal injections (including both the CS and HA injections) on the basis of the literature. The absolute contraindications to intraarticular injections are: hypersensitivity to CS or any other type of injectable substance, systemic infection or infection in the area of the planned injection (cellulitis, septic arthritis, septic bursitis, osteomyelitis), uncontrolled bleeding disorder, prosthetic or unstable joint and finally intraarticular fracture [48]. The relative contraindications to intraarticular injections are: treated bleeding disorder, hemarthrosis, anticoagulants, immunosuppression, diabetes, increased risk of tendon rupture and pain of psychogenic origin [48].

CS are known to cause several effects on articular cartilage. They alter cartilage matrix metabolism [49], change the mechanical properties of articular cartilage [50] and lead to chondrotoxicity [51]. CS intraarticular injections should not be repeated more than four times per year due to the increased risk of articular cartilage destruction. CS injections simultaneously reduce the pain within the joint and remove effusions around the joint. Although the joint pain reduction appears quickly, it does not last long [36].

### 3.3. Platelet-Rich Plasma (PRP)

PRP is an autologous concentrate of platelets and growth factors, derived from centrifugated blood [52,53,54]. There have been listed in the literature two other types of platelet concentrates, namely platelet-rich fibrin (PRF) and plasma rich in growth factors (PRGF) [55]. PRP may be obtained only from the liquid blood. It is impossible to obtain PRP from serum or clotted blood [56].

There are several different commercial protocols to collect blood and to obtain PRP. The differences among them include: the required amount of blood to be taken from the patients, the isolation method, the speed of centrifugation, the amount of obtained concentrated volume after centrifugation, processing time, increase in platelets and platelet capture efficiency [57]. Moreover, it has also been found that different methods of blood centrifugation affect the leukocyte ratios [58].

The number of platelets in 1 µL of blood in healthy individuals ranges from 150,000 to 300,000 [59]. Platelets are responsible for hemostasis and wound healing [52,59]. There are three types of organelles within the platelets: α-granules, dense granules and lysosomes [59]. Alpha granules are the most common ones (approximately 80 granules per cell) [60]. They contain several different types of proteins, including growth factors (i.e., transforming growth factor β, insulin-like growth factor, epidermal growth factor), chemokines, coagulants, anticoagulants, fibrinolytic proteins, adhesion proteins, integral membrane proteins, immune mediators, angiogenic factors and inhibitors and microbicidal proteins [56,59].

The exact mechanism of PRP action remains unclear and is even sometimes questioned [61,62]. It is speculated that PRP enhances wound healing because of the presence of various cytokines, including growth factors. PRP also presents hemostatic properties. Moreover, it may indirectly activate macrophages via serotonin and histamine release, which increase the capillary permeability, consequently leading to the inflammatory cells’ access [56,61,62]. Finally, PRP in in vitro studies has been found to stimulate the chondrocytes to engineer the cartilage and the biosynthesis of collagen and proteoglycans [63].

PRP has been used in various medical specialties, including oral and maxillofacial surgery, dermatology, ophthalmology, cardiothoracic surgery and plastic surgery, but also in the treatment of musculoskeletal disorders, including TMJ OA [61,64,65].

### 3.4. HA, CS and PRP in the Treatment of TMJ OA

HA, CS and PRP may be injected intraarticularly independently as sole, less invasive, surgical procedures or may be injected at the end of other surgical procedures, including arthrocentesis or arthroscopy [66,67,68,69,70,71,72,73,74,75,76,77,78,79,80,81].

Arthrocentesis is a minimally invasive surgical procedure, which aims to eliminate the inflammatory mediators from the inside of the TMJ and to disrupt any adhesions within the TMJ. Either physiological solution or Ringer’s solution is used for the arthrocentesis. Arthrocentesis is performed most often under local anesthesia [8,66].

Arthroscopy, compared to arthrocentesis, is a more invasive surgical procedure, which is predominantly performed under general anesthesia. Arthroscopy requires at least two different ports. This technique is used not only for the intraarticular operations, but also for the real-time visualization of the TMJ [8,80]. Fernández Sanromán et al. [80] used arthroscopy to record, among others, hypervascularization of retrodiscal tissues, articular disc perforations, synovitis, intraarticular areas of hyperemia, presence of intraarticular adhesions and finally areas of bone exposure. 

Although there have been published many studies related to the intraarticular supplementation of HA, CS or PRP, only a few of them are randomized clinical trials (RCTs) that have been performed among patients diagnosed with TMJ OA.

Bergstrand et al. [66] compared the effectiveness of arthrocentesis alone with arthrocentesis combined with an additional injection of HA in the treatment of TMJ OA. The authors presented the most long-term observations (almost 4 years) compared to other RCTs. Bergstrand et al. assessed pain symptoms and jaw function on the basis of the maximum incisor opening, both-side lateral function and mandibular protrusive movement. The authors also analyzed the presence of joint sounds. Both methods led to a significant improvement in jaw function and significant reduction in pain intensity. None of the methods significantly improved joint sound. Both methods were equally effective in the treatment of TMJ OA. Therefore, supplementary injection of HA at the end of arthrocentesis did not improve the final outcome.

Guarda-Nardini et al. [67] compared the efficacy of cycle of 5 single-needle arthrocenteses combined with HA of different molecular weights. The authors injected intraarticularly either LMW HA (Hyalgan) or MMW HA (Sinovial). There were no significant differences between the examined groups, regarding pain at chewing, pain at rest, chewing efficiency, mouth opening and functional limitation. These results indicate that the molecular weight of HA does not affect the efficacy of the TMJ OA treatment. 

Contrary to the previously presented studies, Tang et al. [68] did not combine intraarticular injections of HA with arthrocentesis. They compared the results obtained after five intraarticular injections of either HA or physiologic saline solution. The authors found that only patients treated with HA injections presented significant pain reduction in the area of the TMJs. This study indicates that HA may be effective in pain reduction in the treatment of TMJ OA, when arthrocentesis has not been performed and therefore inflammatory mediators have not been flushed out of the joint.

Bouloux et al. [69,70] compared three groups of patients with TMJ OA treated with arthrocentesis combined with the supplementary injection of HA, CS or Ringer’s solution. The authors assessed efficiency in pain reduction, changes in quality of life, jaw function and maximum incisal opening. Despite the fact that the group which was supplemented with CS presented the lowest improvement in pain reduction, the obtained results were still statistically significant. Furthermore, there were no significant changes between the examined groups regarding jaw function, as well as maximum incisal opening with and without pain. The authors concluded that all three methods of treatment are equally effective in pain reduction, improving jaw function and maximum incisal opening in patients diagnosed with TMJ OA and that additional CS or HA injection does not provide any benefits. Moreover, none of the presented methods led to a significant improvement in patients’ quality of life.

Huddleston Slater et al. [71] assessed the differences in clinical results between arthrocentesis with an additional single-dose injection of isotonic saline 1 cc and arthrocentesis with an additional single-dose injection of CS 1cc. There were no significant differences between the examined groups regarding pain complaints, maximal interincisal opening, as well as functional impairment.

Manfredini et al. [72] compared six different protocols of arthrocentesis with or without supplementary drugs, namely single-session two-needle arthrocentesis, single-session two-needle arthrocentesis plus CS, single-session two-needle arthrocentesis plus LMW HA, single-session two-needle arthrocentesis plus HMW HA, five-weekly two-needle arthrocenteses plus LMW HA and five-weekly single-needle arthrocenteses plus LMW HA. The group of patients treated with single-session two-needle arthrocentesis plus HMW HA was withdrawn from the study because of the severe side effects occurring in two patients. The authors did not find any significant differences among the examined groups regarding changes in the pain at rest, pain at chewing, chewing efficiency and mouth opening.

Bjørnland et al. [73] and Møystad et al. [74] compared the efficacy of two intraarticular injections of either HA or CS in patients diagnosed with TMJ OA. The injections were performed 14 days apart. Bjørnland et al. [73] noticed that only patients treated with HA presented: significantly less pain intensity after 6-month observation and significantly fewer joints with crepitation after first injection. However, there were no significant differences in the improvement of the jaw function, as well as in the presence of the TMJ sounds between the examined groups. Results by Bjørnland et al. [73] support the observations described by Tang et al. [68] regarding the changes in pain intensity after the intraarticular HA injection without simultaneous arthrocentesis. Contrary to Bjørnland, Møystad et al. [74] assessed the presence of the radiographic signs of OA and the progression or regression of osseous changes in the TMJs. The authors did not find any significant radiographic changes between the examined groups.

Isacsson et al. [75] determined the efficacy of a single-dose intraarticular injection of methylprednisolone (CS) and the efficacy of a single-dose intraarticular injection of sodium chloride. There were no statistically significant differences between the examined groups regarding the pain reduction and the function of the mandible. Both groups presented significant pain release and significant improvement in jaw function. However, patients treated with CS developed adverse events related to the treatment more often compared to the patients treated with sodium chloride. Moreover, the injection of methylprednisolone caused increased pain during the first few days after the intervention.

Cömert Kiliç et al. [76] assessed the differences between the clinical outcomes of arthrocentesis performed alone and arthrocentesis with an additional injection of CS. Both methods of treatment led to a statistically significant pain reduction and significant reduction in joint sounds. Only patients who received an additional CS injection presented a statistically significant increase in painless interincisal opening. Comparison of both groups regarding masticatory efficiency, pain complaints, joint sound, painless mouth opening, maximum mouth opening, lateral motion and protrusive motion revealed no statistically significant differences. An additional CS injection did not improve the clinical results.

Apart from the previously described study, Cömert Kiliç et al. [77] compared other methods of treatment of TMJ OA, namely arthrocentesis with lavage and an additional injection of HA and arthrocentesis with lavage and an additional injection of PRP with four consecutive PRP injections (1 per month). The authors found no significant differences between the clinical results obtained in both examined groups. According to the presented study, arthrocentesis with multiple PRP injections was not superior to arthrocentesis with a single HA injection. Finally, Cömert Kiliç et al. [78] also compared the results of arthrocentesis alone with the clinical outcomes of arthrocentesis with lavage and an additional injection of PRP with four consecutive PRP injections (1 per month). Both groups presented a significant reduction in general pain and joint sounds. Moreover, only patients treated with PRP presented significantly increased masticatory efficiency, painless mouth opening and lateral movements after the end of the treatment. However, the only significant difference in clinical outcomes between the groups was related to the masticatory efficiency; specifically, masticatory efficiency was significantly higher in the PRP group. In the authors’ opinion, arthrocentesis with PRP is superior compared to arthrocentesis alone.

Hegab et al. [79] also assessed the clinical outcomes after two different combinations of arthrocentesis with either PRP or HA injections. They treated the patients with either three autologous intraarticular injections of 1 mL of PRP once per week for three consecutive weeks after arthrocentesis with 50 mL of lactated Ringer’s solution or with three intraarticular injections of 1 mL of LMW HA once per week for three consecutive weeks after arthrocentesis was performed in the same way. After 12-month follow-up, patients treated with PRP presented better clinical outcomes in terms of pain reduction and increased interincisal distance than those who had been treated with LMW HA. However, up to 6 months after the end of the treatment, the PRP group presented significantly increased pain and significantly decreased maximum mouth opening compared to the LMW HA group. The authors found that between the 6th and 12th month after the end of the treatment, the LMW HA group presented a significant decrease in median maximum mouth opening and significant increase in the median pain score. The authors concluded that PRP injections lead to better clinical outcomes than HA. However, they also noticed that patients treated with PRP intraarticular injections presented significantly more complications, including pain during injection, as well as postoperative discomfort. 

Finally, there have been published two articles related to arthroscopy [80,81]. Fernández Sanromán et al. [80] performed either arthroscopy with PRGF injection 2 mL or arthroscopy with 5% sodium chloride injection 5 mL in patients diagnosed with Wilkes stage IV internal derangement. Patients treated with an additional PRGF injection presented significantly lower pain scores only 6 and 12 months after the end of the treatment compared to the control group. There were no significant differences between the examined groups regarding pain complaints and maximum mouth opening after 2-year follow-up. The authors indicated that additional PRGF supplementation did not improve the final outcomes. Fernández-Ferro et al. [81] also compared arthroscopy with PRGF injection 5 mL (not 2 mL, as performed by Fernández Sanromán et al. [80]) and arthroscopy with HMW HA injection. Despite the fact that both groups presented a progressive increase in mouth opening, there were no significant differences between the groups. However, PRGF following arthroscopy was more effective than the injection of HA regarding pain control.

Table 3 presents the effectiveness of HA, CS and PRP in the treatment of TMJ OA on the basis of the literature [66,67,68,69,70,71,72,73,74,75,76,77,78,79,80,81].

The above-listed studies differ in methodology, endpoints and obtained results. Many of these studies do compare different protocols of the TMJ OA treatment with the usage of additional intraarticular injections, but at the same they do not compare the obtained results to control groups, which should involve arthrocentesis performed alone. Although the authors find different methods effective, they should also state if the observed methods of treatment are in fact superior to arthrocentesis performed alone. This is important not only for clinical reasons but also for economic ones. The studies that compared different methods of treatment to arthrocentesis alone showed no significant differences regarding the obtained results.

## 4. Conclusions

Arthrocentesis alone effectively reduces pain and improves jaw function in patients diagnosed with TMJ OA. Additional injections of HA (either LMW HA or HMW HA) or CS at the end of the arthrocentesis do not improve the final clinical outcomes. When arthrocentesis is not performed, the intraarticular injection of HA is more effective in pain reduction compared to injections of either CS or physiologic saline solution. Moreover, it seems that intraarticular injections should be repeated more than once to achieve satisfactory clinical outcomes and therefore the number of intraarticular injections should be further evaluated.

However, it should also be noted that CS directly affect the articular cartilage by altering cartilage matrix metabolism, changing the mechanical properties of the articular cartilage and by leading to chondrotoxicity. Because of the fact that CS do not present any superior effects compared to HA or arthrocentesis either alone or combined with HA, the usage of CS in the treatment of TMJ OA should not be recommended and should be further examined.

Results related to additional PRP injections are not consistent and are rather questionable. It seems that PRP injections do not add any significant improvements to maximum mouth opening, but they may effectively reduce pain. The studies regarding the efficacy of intraarticular injections of PRP should be further evaluated. The amount of PRP injected intraarticularly and the number of injections seem to have an impact on the final clinical outcomes.

It is recommended for further studies to include always the control group of patients treated with arthrocentesis alone. It seems that this minimally invasive surgical procedure is enough to reduce the TMJ pain and to satisfactorily increase the maximum mouth opening by flushing out the inflammatory mediators from the inside of the TMJ. Further studies should be multicenter, based on a larger group of patients and should definitively answer the question of whether other methods of TMJ OA treatment, especially those which require the usage of additional intraarticular supplements, are in fact more beneficial for the patients than simple arthrocentesis.

## Figures and Tables

**Table 1:** Inclusion and exclusion criteria for the systematic review.

Criteria	List of Specific Criteria
Inclusion criteria	- randomized controlled trials - randomized clinical trials - study population: adolescents (aged: 16 years old or more) and adults diagnosed with TMJ OA - methods of treatment: arthrocentesis or arthroscopy with an additional injection of HA, CS and PRP or intraarticular injections of HA, CS and PRP - papers written in English
Exclusion criteria	- case–control studies - case reports - comments - systematic reviews and metanalyses - usage of animal models - study population: children under 16 years old and patients diagnosed with other types of TMD without concomitant TMJ OA - methods of treatment: conservative methods of treatment (including physiotherapy, occlusal splint therapy and pharmacotherapy) and invasive surgical procedures (open joint surgery) - papers written in languages other than English

TMJ OA—temporomandibular joint osteoarthritis, HA—hyaluronic acid, CS—corticosteroids, PRP—platelet-rich plasma, TMD—temporomandibular joint disorder.

**Table 2:** Examples of CS injected intraarticularly on the basis of the literature [37,38].

Ester Preparations (Insoluble in Water)	Non-Ester Preparations (Soluble in Water)
Methylprednisolone acetate	Dexamethasone sodium phosphate
Betamethasone acetate	Betamethasone sodium phosphate
Triamcinolone acetonide	
Hydrocortisone acetate	

**Table 3:** Effectiveness of HA, CS and PRP used in the treatment of the TMJ OA on the basis of the literature [66,67,68,69,70,71,72,73,74,75,76,77,78,79,80,81].

References	Study Design	Participants and Intervention	Endpoint and Results
Bergstrand et al. (2019) [66]	Randomized, double-blind study	37 patients (30 women, 7 men, aged 23–83 years): - arthrocentesis with lavage alone (17 patients) - arthrocentesis with lavage and an additional injection of HA (20 patients) - all of the patients received conservative therapies before enrollment in the study (education, non-steroidal anti-inflammatory drugs, physiotherapy, occlusal splints)	Endpoint: 47 months (range: 25–79 months) No significant differences regarding maximum incisor opening and pain reduction between the examined groups. Additional HA injection did not improve the final outcome.
Guarda-Nardini et al. (2012) [67]	Randomized, double-blind study	35 patients (30 women, 5 men, mean age—group A: 47.7 ± 15.0 years; group B: 52.9 ± 16.1 years): - group A: arthrocentesis + 1 mL of medium-molecular-weight HA (17 patients) - group B: arthrocentesis + 1 mL of low-molecular-weight HA (18 patients) - all of the patients underwent a cycle of 5 single-needle arthrocenteses with injection of 1 mL of HA (1x/week)	Endpoint: 3 months No significant differences between the examined groups regarding the effectiveness of both methods of treatment.
Tang et al. (2010) [68]	Randomized, double-blind study	40 patients (21 women, 19 men, aged: 25–63 years): - SH group: 5 injections of sodium hyaluronate 1 mL once a week for 5 weeks (20 patients) - control: 5 injections of physiologic saline solution 1 mL once a week for 5 weeks (20 patients)	Endpoint: after 5-week treatment Only patients treated with SH presented significant pain reduction.
Bouloux et al. (2017) [69,70]	Randomized, double-blind study	102 patients (89 women, 13 men, mean age—group CS: 39.6 ± 18.4 years; group HA: 44.3 ± 17.2 years; group Ringer: 51.8 ± 17.2 years): - arthrocentesis with lavage and an additional injection of CS 1 mL (35 patients) - arthrocentesis with lavage and an additional injection of HA 1 mL (36 patients) - arthrocentesis with lavage and an additional injection of Ringer’s solution 1 mL (31 patients)	Endpoint: 3 months No significant differences among the examined groups regarding pain levels, maximum incisal opening, jaw function and quality of life.
Huddleston Slater et al. (2012) [71]	Randomized, double-blind study	28 patients (23 women, 5 men, mean age—control group: 33.9; group CS: 32.6): - control: arthrocentesis with an additional single-dose injection of isotonic saline 1 cc (14 patients) - CS group: arthrocentesis with an additional single-dose injection of CS 1 cc (14 patients)	Endpoint: 24 weeks No significant differences between the examined groups regarding pain complaints, maximal interincisal opening, as well as functional impairment.
Manfredini et al. (2012) [72]	Randomized, single-blind study	60 patients (51 women, 9 men, mean age 50.1 years) - protocol A: single-session two-needle arthrocentesis (11 patients) - protocol B: single-session two-needle arthrocentesis plus CS (9 patients) - protocol C: single-session two-needle arthrocentesis plus LMW HA (11 patients) - protocol D: single-session two-needle arthrocentesis plus HMW HA (5 patients) because of the severe side effects occurring in two patients, the group was withdrawn from the study - protocol E: 5 weekly two-needle arthrocenteses plus LMW HA (12 patients) - protocol F: 5 weekly single-needle arthrocenteses plus LMW HA (12 patients)	Endpoint: 6 weeks No significant differences among the examined groups regarding the pain at rest, pain at chewing, chewing efficiency and mouth opening.
Bjørnland et al. (2007) [73]	Randomized, double-blind study	40 patients (34 women, 6 men, mean age—group HA: 53.4 ± 12.9 years; group CS: 50.0 ± 13.3 years) - S-group: two intraarticular injections 14 days apart with 0.7–1 mL of HA (20 patients) - C-group: two intraarticular injections 14 days apart with 0.7–1 mL of CS (20 patients)	Endpoint: 6 months Patients treated with HA presented: - significantly less pain intensity after 6 months - significantly fewer joints with crepitation after first injection - There were no significant differences in the improvement of jaw function or in the presence of TMJ clicking between the examined groups.
Møystad et al. (2008) [74]	Randomized, double-blind study	36 patients (31 women, 5 men, mean age—group HA: 51.5 ± 12.9 years; group CS: 48.3 ± 13.5 years) - S-group: two intraarticular injections 14 days apart with HA (17 patients) - C-group: two intraarticular injections 14 days apart with CS (19 patients)	Endpoint: 6 months No significant differences between the examined groups regarding the presence of the radiographic signs of osteoarthritis or regarding the progression or regression of osseous changes in the TMJs.
Isacsson et al. (2019) [75]	Randomized, double-blind study	54 patients (44 women, 10 men, mean age—group A: 48 ± 18.6 years; group B: 56 ± 14.7 years): - group A: 1 mL intraarticular injection of methylprednisolone 40 mg/mL (27 patients) - group B: 1 mL intraarticular injection of sodium chloride (27 patients) - all of the patients received a single-dose intraarticular injection	Endpoint: 4 weeks No significant differences between the examined groups regarding TMJ arthralgia pain reduction. Methylprednisolone led to increased pain following the intervention compared to saline.
Cömert Kiliç et al. (2016) [76]	Randomized clinical trial	24 patients (21 women, 3 men, mean age—control group: 35.08 ± 14.84 years; group CS: 32.58 ± 9.58 years): - control: arthrocentesis (12 patients) - CS group: arthrocentesis with an additional single-dose injection of CS 1 mL (12 patients)	Endpoint: 12 months No significant differences between the examined groups regarding pain complaints or range of motion.
Cömert Kiliç et al. (2016) [77]	Randomized clinical trial	31 patients (26 women, 5 men, mean age: 30.48 ± 13.04 years): - PRP group: arthrocentesis with lavage and an additional injection of PRP 1 mL + 4 consecutive PRP injections (1 per month) (18 patients) - HA group: arthrocentesis with lavage and an additional injection of HA 1 mL (13 patients)	Endpoint: 12 months No significant differences between the examined groups regarding masticatory efficiency, pain complaints, joint sounds, painless mouth opening, maximum mouth opening, lateral and protrusive movement.
Cömert Kiliç et al. (2015) [78]	Randomized clinical trial	30 patients (27 women, 3 men, mean age—control group: 35.08 ± 14.84 years; group PRP: 32.22 ± 14.33 years): - control: arthrocentesis (12 patients) - PRP group: arthrocentesis with lavage and an additional injection of PRP 1 mL + 4 consecutive PRP injections (1 per month) (18 patients)	Endpoint: 12 months No significant differences between the examined groups regarding pain complaints, joint sounds, painless mouth opening, maximum mouth opening, lateral and protrusive movement. Only masticatory efficiency was significantly higher in PRP group.
Hegab et al. (2015) [79]	Randomized single-blind study	50 patients (29 women, 21 men, aged 31–49 years): - PRP group: 3 autologous intraarticular injections of 1 mL of PRP once per week for 3 consecutive weeks after arthrocentesis with 50 mL of lactated Ringer’s solution (25 patients) - HA group: 3 intraarticular injections of 1 mL of LMW HA once per week for 3 consecutive weeks after arthrocentesis with 50 mL of lactated Ringer’s solution (25 patients)	Endpoint: 12 months PRP performed better than LMW HA during long-term follow-up (12 months) in terms of pain reduction and increased interincisal distance (up to 6 months better results were obtained with LMW HA).
Fernández Sanromán et al. (2016) [80]	Randomized single-blind study	92 patients (87 women, 5 men, aged 17–67 years): - PRGF group: arthroscopy + PRGF injection 2 mL (42 patients) - control group: arthroscopy + 5% sodium chloride injection 5 mL (50 patients)	Endpoint: 2 years No significant differences between the examined groups regarding pain complaints and maximum mouth opening.
Fernández-Ferro et al. (2017) [81]	Randomized single-blind study	100 patients (94 women, 6 men, aged 18–77 years): - PRGF group: arthroscopy + PRGF injection 5 mL (50 patients) - control group: arthroscopy + HMW HA injection (50 patients)	Endpoint: 18 months No significant differences between the examined groups regarding maximum mouth opening. PRGF following arthroscopy was more effective than the injection of HA regarding pain control.

TMJ—temporomandibular joint, HA—hyaluronic acid, SH—sodium hyaluronate, CS—corticosteroids, PRP—platelet-rich plasma, PRGF—plasma rich in growth factors, LMW HA—low-molecular-weight hyaluronic acid, HMW HA—high-molecular-weight hyaluronic acid.

## Data Availability

The data underlying this article are available in the article.
